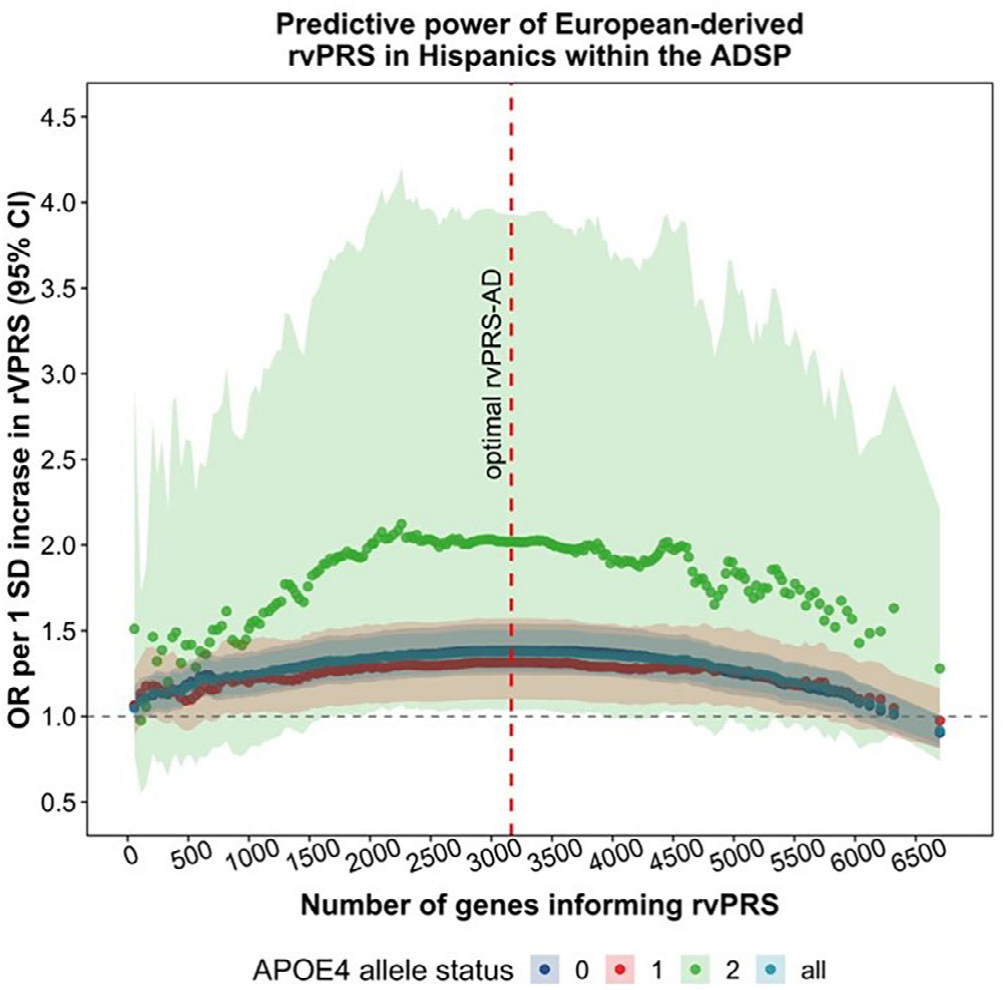# A rare variant polygenic risk score for Late‐Onset Alzheimer's Disease demonstrates trans‐ethnic predictive power

**DOI:** 10.1002/alz70855_106838

**Published:** 2025-12-25

**Authors:** Ricky Lali, Shihong Mao, Basilio Cieza, Guillaume Pare, Giuseppe Tosto

**Affiliations:** ^1^ Department of Pathology and Molecular Medicine, McMaster University, Hamilton, ON, Canada; ^2^ department of pathology and molecular medicine, hamilton, ON, Canada; ^3^ Columbia University, New York, NY, USA; ^4^ Gertrude H. Sergievsky Center, Taub Institute for Research on the Aging Brain, Departments of Neurology, Psychiatry, and Epidemiology, College of Physicians and Surgeons, Columbia University, New York, NY, USA; ^5^ Taub Institute for Research on Alzheimer's Disease and the Aging Brain, Vagelos College of Physicians and Surgeons, Columbia University, New York, NY, USA; ^6^ Department of Neurology, College of Physicians and Surgeons, Columbia University, and the New York Presbyterian Hospital, New York, NY, USA

## Abstract

**Background:**

Late‐onset Alzheimer's Disease (LOAD) is a substantial contributor to global morbidity, with cases expected to triple by 2050. While aging is the strongest environmental risk factor, genetic factors account for over 60% of disease variation. Traditional polygenic risk scores that aggregate an individual's genetic risk aid in risk stratification, but rely solely on common variants, which limit predictive power across populations, reducing generalizability of genetic risk. The gene‐based burden of rare damaging variants has never been assessed for LOAD in a polygenic framework, despite its predictive potential across ancestral groups.

**Method:**

We herein develop the first rare variant polygenic risk score for LOAD (rvPRS‐AD) using a whole genome‐adapted version of RV‐EXCALIBER, a method that implements correction factors to calibrate rare‐variant gene burden testing against large, summary‐level control data from gnomAD. Using 3,842 European LOAD cases from ADSP and 32,299 non‐Finnish Europeans from gnomAD as controls, we identified 3,164 risk‐conferring genes (excluding *APOE*), which were used to construct rvPRS‐AD in 2,433 Hispanic ADSP participants (671 LOAD cases, 1,762 controls) by weighting the additive burden of rare damaging variants per gene.

**Result:**

We identified *ABCA7*, a well‐established Alzheimer's risk gene, as the most strongly associated gene with LOAD (OR = 1.40, *p* = 1×10⁻⁴). rvPRS‐AD demonstrated trans‐ancestral predictive power, with a 1‐SD increase in the European‐derived score conferring 38% higher odds of LOAD (95% CI, 1.26–1.51) among Hispanics (Figure 1). It remained predictive independent of *APOE*ε4 status and was strongest among *APOE*ε4 homozygotes (OR = 2.02, 95% CI, 1.04‐3.93). Lastly, rvPRS‐AD identifies individuals at extreme LOAD risk, with 2.2% of Hispanic participants exhibiting a ≥2‐fold increased odds of disease.

**Conclusion:**

rvPRS‐AD is the first genetic instrument that leverages rare variants in a polygenic framework to predict LOAD risk across ancestrally diverse populations in a manner that is independent of common variant predictors.